# Supplementary figures and images for: Relative telomere length and oxidative DNA damage in hypertrophic ligamentum flavum of lumbar spinal stenosis
Source: PeerJ. 2018 Aug 9;6:e5381. doi: 10.7717/peerj.5381 (PMC6087619; doi:10.7717/peerj.5381)

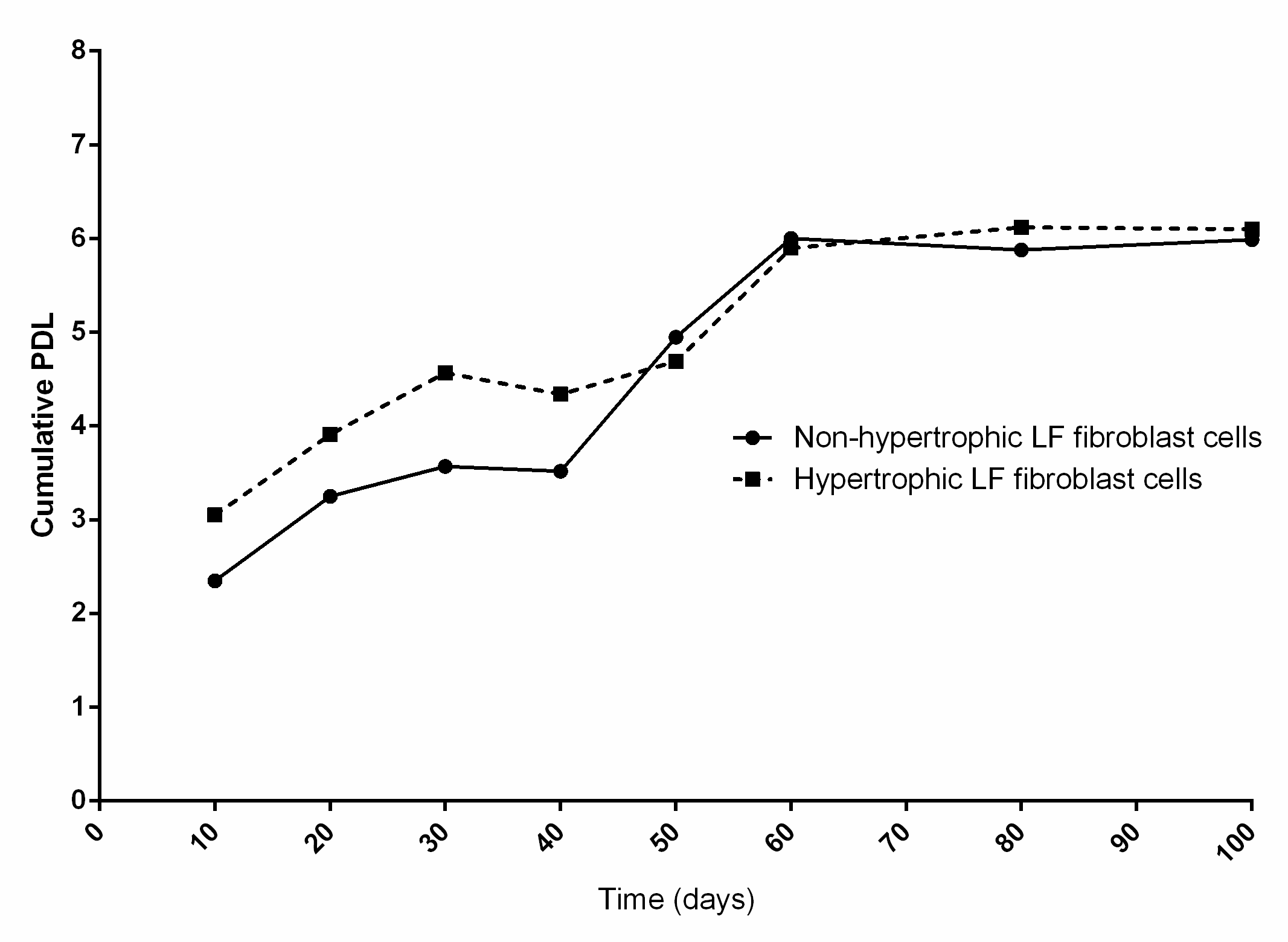

Supplement: Figure S1 — The cumulative PDL of hypertrophic and non-hypertrophic LF cells from patient aged 66 years. Human hypertrophic and non-hypertrophic LF cells were serially cultivated for 100 days until the cells reached the end of their lifespans. [file peerj-06-5381-s002.png]

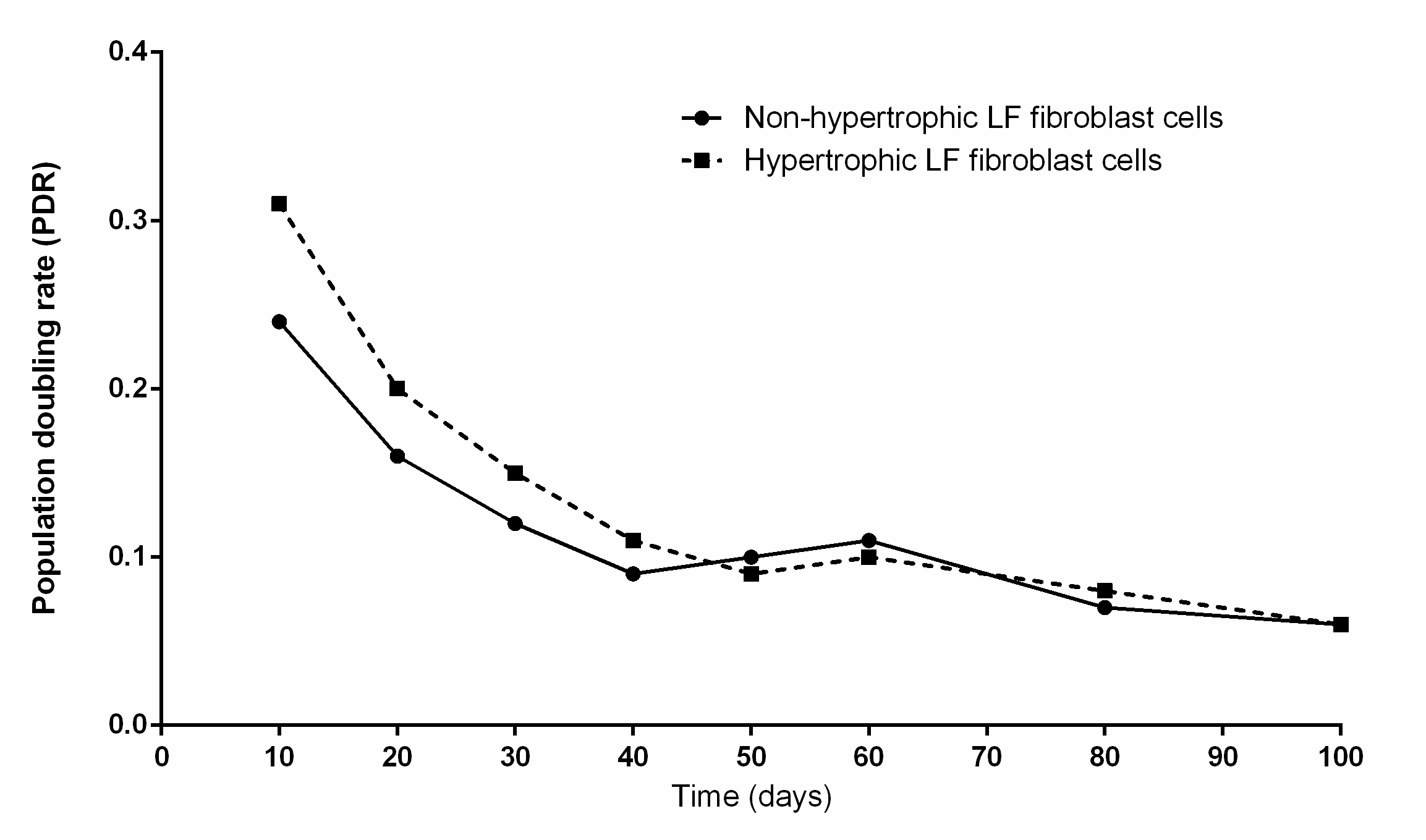

Supplement: Figure S2 — Growth curves of human hypertrophic and non-hypertrophic LF cells showing PD rate from patient aged 66 years in cell culture. [file peerj-06-5381-s003.png]
